# The Epidemiology of COVID-19 in the Gansu and Jinlin Provinces, China

**DOI:** 10.3389/fpubh.2020.555550

**Published:** 2020-09-11

**Authors:** Jingchun Fan, Brett D. Hambly, Shisan Bao

**Affiliations:** ^1^School of Public Health, Gansu University of Chinese Medicine, Lanzhou, China; ^2^Center for Evidence-Based Medicine, Gansu University of Chinese Medicine, Lanzhou, China; ^3^Discipline of Pathology, Faculty of Medicine and Health, The School of Medical Sciences, The University of Sydney, Sydney, NSW, Australia

**Keywords:** SARS-CoV-2, China, COVID-19, second wave, primary wave

## Abstract

The COVID-19 outbreak has become a pandemic. The outbreak was able to be controlled in China by mid-April through the implementation of critical measures; however, significant reverse transmission has resulted in hot spots perturbing prevention and control. To date, there have only been a total of 92 indigenous COVID-19 cases confirmed in the Gansu Province, which is considered to be a consequence of the strict screening approach applied during the outbreak. The emergency response level to COVID-19 were able to be decreased from high to low, despite some relatively minor reverse transmission cases from other countries in March 2020. The stringent preparative measures undertaken by the Gansu authorities, involving high-level, streamlined cooperation between the transportation, quarantine, and medical resource departments, have underpinned this success. There has been an emergence of clusters of freshly infected COVID-19 patients in the Jilin Province in northeast China. The single largest cluster has been in Shulan of the Jilin Province, involving 43 confirmed infections. A strict lockdown was implemented immediately. The source of the current outbreak of COVID-19 is suggested to be travelers returning from Russia. The current strategy from the Chinese authorities is aimed at preventing reverse transmission via international importation to avert a rebound of COVID-19 in China. These data highlight the need for an exceptionally high level of vigilance and for a pre-emptive response that is informative for the development of policy to prevent a second and further waves of infections in general.

## Introduction

2019 novel coronavirus disease (COVID-19), caused by infection with SARS-CoV-2 virus, was originally discovered in Wuhan, Hubei Province of Central China in December 2019 (1). The outbreak of COVID-19 was far more severe than anyone expected due to insufficient knowledge of the SARS-CoV-2 virus transmission during the initial stages of the spread (2). Currently (as at August 12, 2020), data on the extent of the pandemic are as follows: the pandemic has involved 215 countries and territories with a total of 19,936,210 confirmed cases that have been reported, including a total of 732,499 deaths (3). Wuhan is located in Central China with a population of 15 million (4). Due to the impending Chinese New Year, more than 5 million people traveled from Wuhan for either family reunions and/or holidays (5), contributing to the subsequent outbreak of COVID-19 in every province/region in China within a matter of weeks (6) that evolved into a pandemic within a matter of months (7). In response to the spread of the virus, a strict lockdown was implemented in late January 2020 in China in an attempt to stop person-to-person transmission, including the mandatory use of face masks in public, no public gatherings, and school and factory closures (8). It has been striking to observe that these measures were able to substantially reduce the number of COVID-19 cases to close to zero within a month, i.e., by February 2020 (9). In addition, mandatory COVID-19 testing was instigated for all staff and patients in every in-patient department in all hospitals, including accompanying family members (10). As expected, subsequently there have been almost no new COVID-19 cases reported in China (11). Since late April 2020, almost all schools in China have been allowed to re-open, following initial online teaching only during March and April 2020 (12). Manufacturing industries around the country have gradually reopened following the reopening of schools (13). This evidence supports the remarkable achievement in controlling the outbreak of COVID-19 within China (14).

While most publications by clinicians and researchers have been focusing on the epicenter of COVID-19, i.e., Wuhan, China, this manuscript aims to cover the epidemiology of the COVID-19 infection in the Northern region of China, namely the Gansu (Northwest) and Jilin (Northeast) Provinces. The primary outbreaks in the Gansu and Jilin Provinces were very similar and mild during the first wave that occurred in January and February 2020. However, once the initial outbreak was under control, Gansu accepted the task of quarantining Chinese nationals returning from abroad and undertook to provide treatment for those returnees who were infected with coronavirus. On the other hand, Jilin subsequently experienced a second wave of infection triggered by asymptomatic cases. In this review, we will outline the differences in the epidemiological approaches adopted by the two provinces in northern China to provide the scientific basis for epidemic prevention and control.

## Importance of the Public Health Response

We hypothesize that the general population continues to face dangerous SARS-CoV-2 viral transmission from distant locations, including from the epicenter (Wuhan, Hubei Province, China), if no effective measures are implemented, despite considerable precautions being undertaken by the provincial governmental authorities. One of the current critical challenges in China is to detect and avert possible reverse transmission of SARS-CoV-2 virus from overseas. The information from our current studies provides some key points that could be used by other regions/countries where COVID-19 is still not yet over the peak of the outbreak.

Recently, we have demonstrated that the primary COVID-19 cases seen in northern China were originally transmitted from Wuhan, Hubei Province, China (2). It is well-documented that COVID-19, originally discovered in Wuhan in late December 2019, was transmitted to Northern China (2). We have reported that within 10 days, from January 23 to February 3, 2020, there were 54 people infected with the SARS-CoV-2 virus, where 35 cases had traveled from Wuhan, and 19 were infected by close contact with the identified travelers. However, the identification of case zero or the index case in northern China could not be made with absolute certainty because the index case in most countries has been found to be asymptomatic (15). Thus, it is critically important to develop novel diagnostic tool(s) with both high sensitivity and specificity to combat this devastating pandemic. Our data suggest that the implementation of adequate interventions has been able to decrease transmission of the COVID-19 virus in the Gansu Province. Following the pandemic of COVID-19 within months of the original outbreak in China, the countries most affected at the time point of March 2020 were Italy (16) and Iran (17). Despite some precautions being undertaken in Italy and Iran in late February 2020, e.g., reducing public gathering and implementing social distancing in Italy and cancellation of mosque worship in Iran and blockage of interstate travel (18), the morbidity and mortality was still able to increase with enormous speed in early March 2020 (19). The increased incidence of COVID-19 in Italy and Iran after emergency response measures were implemented may be due to the long incubation period of the SARS-CoV-2 viral infection, which may be up to 20 days (20). Furthermore, this rapid spread may also be due to relatively low adherence to the restriction orders within these two countries (21). To provide shelter for the overseas Chinese residents in risky countries from the potential risk of COVID-19, the Chinese government provided chartered planes to repatriate these Chinese citizens back to China (22). The destination for these returnees from Italy and Iran was the Zhejiang and Gansu Provinces, respectively (23).

## Primary Outbreak of COVID-19 in the Jilin and Gansu Provinces, China

**Figure 1:**
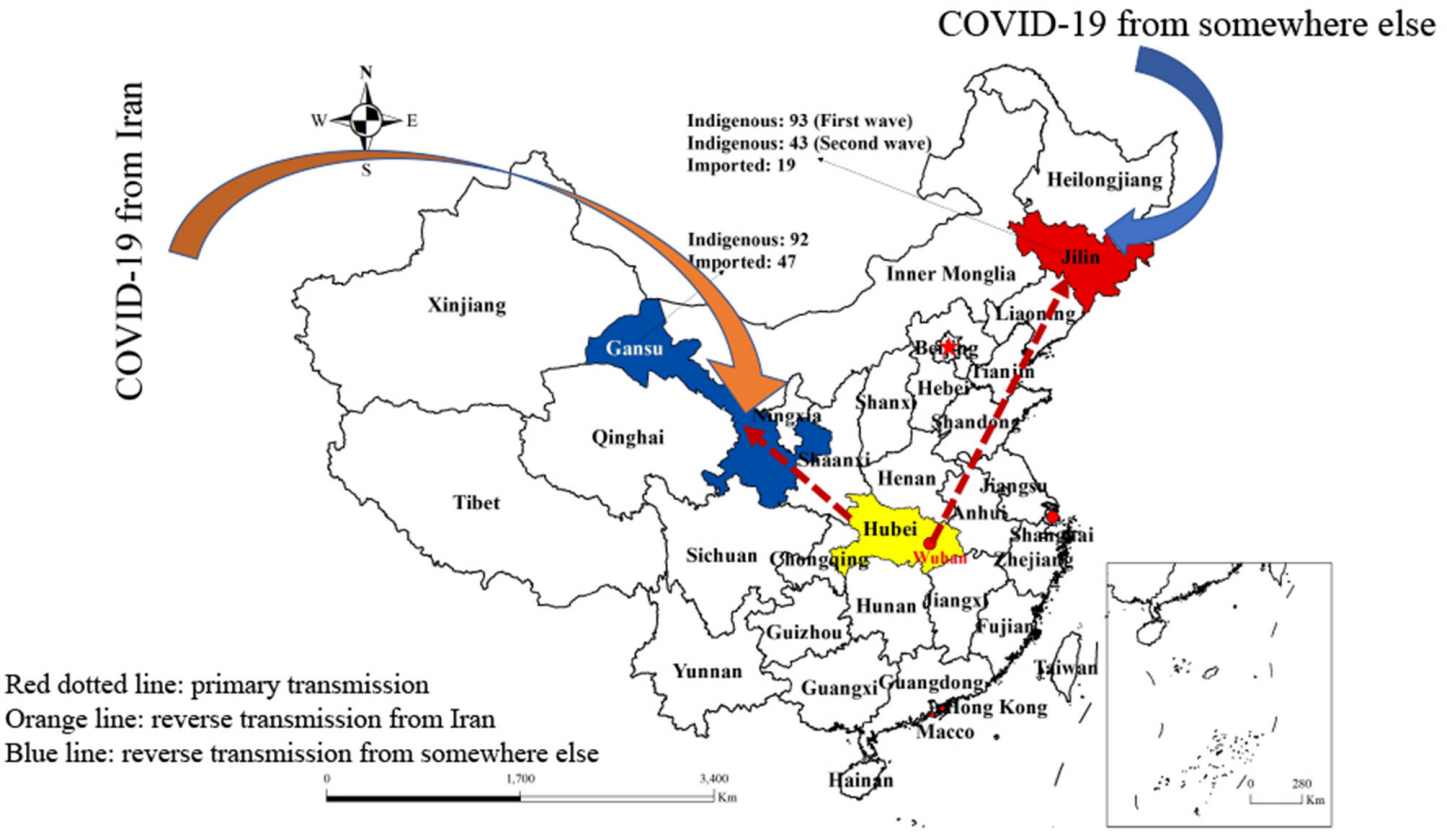
The location of Jilin Province and Gansu Province with the updated indigenous and imported COVID-19 cases. The figure demonstrates the primary COVID-19 transmission from Hubei province to Gansu and Jilin Provinces (in red dotted lines). The secondary reverse transmission from Iran to Gansu Province has been labeled with an orange color, and the same from an unidentified source to Jilin Province is labeled with a blue line.

We have reviewed the epidemiology of COVID-19 in Jilin and Gansu provinces, Northern China. Jilin Province is located in the middle of the northeast of China, covering an area of 187,400 km^2^ with a total population of 27,746,000. Due to the strategically important location in the northeast of China, the Jilin Province is an important gateway connecting the Eurasian land route via Siberia, e.g., the Jilin Province is only 4 km from Vladivostok, Russia, and 15 km from the Sea of Japan (24). On the other hand, the Gansu Province is very similar to the Jilin Province in several aspects. The Gansu Province is located in the northwest of China, covering an area of 454,000 km^2^ with a total population of 26,257,100 (25). Geographically the Gansu Province is also a key transportation hub connecting to five provinces in northwest China. Although the Gansu Province is located in a rather remote region in northwest China, it is considered to be the beginning of the Silk Road (Figure 1). During the primary outbreak, there were 93 and 92 cases, including two deaths, in the Jilin and Gansu Provinces, respectively (26, 27).

## Second Wave: Reverse Transmission of COVID-19

The epidemic of COVID-19 was brought under control in these two provinces in March 2020 and remained under control for almost 2 months. In response to this level of control, the Jilin government proposed a series of measures to control COVID-19. Similarly, within the Gansu Province, the schools have reopened, and the enterprises have been able to recover and re-commence production. During May 2020, however, there has been a re-emergence of COVID-19 in northeast China, particularly in the Jilin Province, while fortunately northwest China has remained free of new infections. It has been reported from the Reuters news agency that another outbreak of COVID-19 has been detected in the Jilin Province, in northeast China, since the removal of the restrictive lockdown and the increase in public activity since April 8, 2020 (28).

A single new case of COVID-19 was discovered on May 7, 2020, in Shulan, in the Jilin Province, without any obvious history of contact with COVID-19 patients and also without a history of interstate/international travel. Additionally, 11 COVID-19 cases were confirmed on May 9, 2020, which broke the record of 73 days of no new cases in this city (26). An immediate lockdown was then implemented in Shulan, Jilin, from May 10, 2020, and the risk level was increased from level II (moderate) to level III (high) (26). The prevention and control measures implemented in Shulan are as high as those in Wuhan at the peak of infection, which was the original epicenter of COVID-19. However, despite the strict lockdown in Shulan, the number of new COVID-19 cases has continued to increase to 43 as of May 20, 2020, which is thought to be a consequence of the close contact of the infected people in this clustering outbreak. It has been reported that the Chinese national returnees coming from Russia have mainly traveled back via train, suggesting that poor screening and quarantine measures may have occurred, and that most imported cases in the Jilin Province are from Russia (26).

In response to the primary COVID-19 outbreak in their Provinces, the authorities from both the Gansu and Jilin Provinces found themselves dealing with this form of emergency for the first time (2). The response included strict prohibition of public gatherings, limitation of social activity to an extremely minimal level, and in-house working via the internet during the outbreak of COVID-19 (29, 30) and these measures were rigorously adhered to. These emergency approaches to deal with COVID-19 were closely modeled on those developed within the epicenter, Wuhan, Hubei Province of China. However, during the reverse transmission outbreak (or second wave), these provincial authorities had already had previous experience in dealing with this subsequent COVID-19 outbreak (22).

Infection in the Gansu Province has been shown to have occurred in two stages. The first stage was the imported case stage of the epidemic, meaning that the cases arrived in travelers from Wuhan (2). In the second indigenous case stage, the patients have been mainly shown to have been infected by the cases from the first imported stage (2). Importantly, during the progression of the COVID-19 epidemic in the Gansu Province, the basic reproduction number (R_0_) has been shown to have decreased from 2.61 in the first stage to 0.66 in the second stage (31), which largely due to the substantially more strict social distancing arrangements implemented during the second stage. New cases of COVID-19 almost reached zero in Northern China within the period from late March 2020 to the middle of May 2020 because of the implementation of restrictive orders. In addition, the clinical interventions for COVID-19 patients were also effective and efficient in reducing morbidity and mortality, in addition to the restrictive quarantine approach (32). Consequently, the mortality rate in Northern China was only two in the Gansu (26) and Jilin Provinces (27).

As the epidemic progressively came under control in China, an alarmingly rapid spread of the virus occurred worldwide, and the epidemic became a pandemic. As the preferred place for receiving Chinese returnees by the Chinese authority, Lanzhou city has received a total of 311 evacuated Chinese citizens from Iran, amongst whom there has been 37 confirmed positive cases of COVID-19 infection, which were only discovered shortly after arrival in Lanzhou (32). Compared to the handling procedures utilized during the primary outbreak of COVID-19 in the Gansu province, the local government had gained substantially increased knowledge and experience in controlling the transmission of SARS-CoV-2 virus that they were able to apply during the secondary reverse transmission of COVID-19 (32). Consequently, due to a substantially more organized level of preparation, local Gansu authorities were able to implement an effective approach in advance of the evacuation, involving high-level, streamlined cooperation among the departments of transportation, quarantine and hospitals, aiming to isolate, and quarantine for 14 days all potentially infected evacuees within designated hotels to prevent the potential risk of transmission of SARS-CoV-2 virus within the Chinese communities of origin of the evacuees. In addition to these organized returnees from Iran, 10 COVID-19 patients have been confirmed in the Gansu Province among independent travelers from abroad who have traveled from locations such as Saudi Arabia and the United States of America (33).

Unfortunately, a proportion of these infected international travelers who returned to China, including to the Jilin and Gansu Provinces, during the early stages of international spread before March 2020 were able to scatter within the community without being quarantined (34), which caused a significant potential risk of the spread of COVID-19. The reason why these COVID-19-infected travelers were able to scatter within their local provinces was that no testing for COVID-19 was undertaken, as COVID-19 testing for returnees was not mandatory in early March 2020—the beginning of the first wave of the outbreak. Subsequently, the local authorities have learnt a heavy lesson from these mistakes and implemented much greater restrictive orders. These data highlight the need for an exceptionally high level of vigilance and the need for a pre-emptive response to prevent a second wave occurring within a community, where the pandemic had been successfully controlled, from returnees from other international locations where the extent of infection at those distant sites had not yet been fully realized.

With the recognition of the seriousness of the SARS-CoV-2 virus in May 2020, the local and central governments called for strengthening of border biosecurity controls, including in the North-eastern provinces, e.g., the Jilin Province, where a growing cluster of infections near the Russian and North Korean borders has threatened to develop into a second wave (35). In addition to the lockdown in the Jilin Province, in order to further reduce possible inadvertent transmission, all private clinics in the Jilin Province have been temporarily suspended until further notice. All patients requiring assessment are now required to attend public hospitals for help, especially for any patients with suspected symptoms associated with COVID-19 who should go to the specialist fever clinics. Thus, Chinese authorities have sought to exhibit flexibility with a rapid response time to enhance the control of the COVID-19 epidemic in key areas that require increasing regular prevention measures in line with the changing situation of the outbreak (36).

One effective approach that has been applied by the Chinese authorities is to launch a health QR (quick response) code system on each individual's smartphone; it is intended to offer a reasonably good indicator within the general population of potential infective status to keep the virus from spreading further. The healthy tracking application has been used previously in monitoring other chronic illnesses for several different purposes (37). This healthy tracking system provides either a green or red code, i.e., non-infected or infected person, respectively. This rating system permits the green code individuals to restart normal activities with minimal risk of infection to others. However, the health QR code system is not foolproof. For example, there has been one individual in Lanzhou with a green code who had traveled from the Hubei Province. A nasal swab RNA test later confirmed that this individual was infected with COVID-19 but asymptomatic (38).

It should be cautioned that there is no significant difference in the secondary infection rate of COVID-19 within the population, caused by infected individuals who are either symptomatic or asymptomatic (39). With this in mind, the Chinese authorities have also been paying particular attention to the detection of asymptomatic cases to prevent further spreading. Interestingly, a comparable project, the Australian Sentinel Practice Research Network (ASPREN) surveillance program, is currently being used for COVID-19 detection in Australia, which was originally intended for monitoring influenza-like illnesses (40). Nevertheless, this approach is in line with an Australian proposal of a system of sentinel testing of people in which large numbers of random, but potentially risky, individuals have been presumptively tested irrespective of showing any symptoms. Such an approach has enabled the authorities to gauge the extent of asymptomatic carriers and detect infection clusters before any infected individuals develop clinical symptoms (41).

Thus, it is essential for the authorities in China to identify these potential COVID-19 risk populations, including local residents and/or overseas returnees, using a more sensitive diagnostic approach, e.g., detection of serum antibodies (42), in addition to nucleic acid testing, which only detects the presence of the virus. Such an approach probably offers greater reliability and flexibility in dealing with potentially infected people within an infection cluster.

## Screening Policy and Controlling Strategies

In Wuhan, the epicenter of COVID-19 infection, a series of policies were implemented. It was confirmed that SARS-CoV-2 virus was able to be transmitted from person-to-person on January 20, 2020. Although COVID-19 was classified as a category B infectious disease, the procedures for preventing and controlling category A infectious diseases (e.g., plague and cholera) were adopted (43). The implementation of these procedures was undertaken by the Wuhan local government, including, firstly, the mandatory wearing of facial masks in a list of public places, including hotels and department stores, and, secondly, strict limitations on outdoor and group activities, particularly in relation to banning public and/or private social gatherings (44). Finally, a complete lockdown of Wuhan was commenced on January 23, 2020, and it lasted for 76 days until April 8, 2020 (45), including a complete shutdown of manufacturing facilities and shops except for essential food and groceries.

Following the concurrent confirmation of the first COVID-19 case on January 23, 2020, in the Gansu and Jilin Provinces (1, 46), the Gansu and Jilin provincial governments immediately implemented the following policies for preventing and controlling COVID-19; emergency response measures were raised to the highest level, effective immediately, which was equivalent to the policies applied in Wuhan at the same time. The emergency response levels to any infectious diseases are classified by the National Health Commission of China (47).

General population screening in China has included mandatory temperature checking for everyone entering any building, using a temperature gun. In addition, for quarantine purposes, monitoring of people's movements was undertaken using a smartphone QR health code system, where an on-screen QR code (for a quick response) was required at the entrance to all buildings to facilitate contact tracing in the event that any positive case was confirmed within the building. Any person with a continuous abnormal temperature was required to have a COVID-19 RNA test for screening confirmation (47). Nevertheless, using this high-level emergency response has proven to be extremely useful, demonstrating that COVID-19 has been effectively brought under control. Consequently, on February 26 and March 2, 2020, the Jilin and Gansu governments lowered the emergency response measures from high to medium and high to low, respectively (48, 49). However, the policy of screening within the general population, i.e., temperature monitoring, and the use of the QR code app are still being used as a major screening approach to the present time (June 2020).

In northern China, the sequential procedures that were adopted were as follows: city lockdown, use of road blocks except for essential travel, maintenance of social distancing, restrictions on social gatherings, mandatory wearing of face masks in public, closure of manufacturing facilities and schools, temperature checking at building entrances, reporting of whereabouts and health condition via QR code app, and remote online working and schooling in the Gansu and Jilin Provinces during the first wave of COVID-19. In response to the second wave in the Jilin Province, the emergency response was immediately re-implemented as described above. The series of strategies implemented to control COVID-19 spreading in Wuhan were essentially the same procedures that were utilized in both the Gansu and Jilin Provinces, the only difference being the commencement and finishing times (Table 1).

**Table 1:** The strategies implemented to control COVID-19 epidemic spread (taking Wuhan as an example).

**No**.	**Strategies**	**Implementation duration**
1	Keep social distance	20 January to 8 April 2020
2	Restrict social gathering	20 January to 8 April 2020
3	Mandatory face mask wearing in public	22 January 2020 till now
4	Suspending production	22 January to 8 April 2020
5	School closure	22 January 2020 till now
6	Temperature checking at building entrance	23 January 2020 till now
7	Lockdown of Wuhan	23 January to 8 April 2020
8	Blockage of unnecessary traveling	23 January to 8 April 2020
9	QR code reporting health condition	23 January to 8 April 2020
10	Internet remote working and schooling	10 February 2020 till now

## Conclusion

In conclusion, COVID-19 is almost completely controlled in the general population of the Gansu Province in northwest China. The first lesson we have learnt from these studies, up to the present time, is that the SARS-CoV-2 virus is able to be transmitted among people very effectively. Thus, it is necessary that strict prohibition of public gatherings, limiting social activity to an extremely minimal level, and remote online working during the outbreak of COVID-19 (29, 30) should be rigorously adhered to. However, it is still debatable whether mandatory wearing of face masks should be undertaken (50, 51). From a public health and safety point of view, it is crucial to continue robust vigilance and implement aggressive control measures to prevent further outbreaks of COVID-19 until complete containment of the pandemic is achieved.

## Limitations

Despite using primary data collected from the local health officials (23), we acknowledge that there are limitations for the current mini review. One limitation is that there has been only one original research paper published concerning the recent and current situation in the Jilin Province (46). Additionally, our available data are insufficient to calculate the R_0_ in the Jilin Province and the R_0_ during the second wave in the Gansu Province, and there has been no published data concerning the R_0_ in these two provinces, which we will determine in our future studies. Notably, the most significant outcomes of the second wave of the outbreak in the Jilin Province are still evolving and hence are not settled yet. This is despite all necessary measures that were used in the control of the first wave of COVID-19 having been implemented.

## Author Contributions

JF and SB have concieved the manuscript and wrote it. BH has editted the manuscript. All authors contributed to the article and approved the submitted version.

## Conflict of Interest

The authors declare that the research was conducted in the absence of any commercial or financial relationships that could be construed as a potential conflict of interest.
